# Periodical Literature

**Published:** 1860-11

**Authors:** 


### HALL'S JOURNAL OF HEALTH.

**Our Legitimate Scope is almost boundless: for whatever begets pleasurable and harmless feelings, promotes Health; and whatever induces disagreeable sensations, engenders Disease.**

WE AIM TO SHOW HOW DISEASE MAY BE AVOIDED, AND THAT IT IS BEST, WHEN SICKNESS COMES, TO TAKE NO MEDICINE WITHOUT CONSULTING A PHYSICIAN.

**Vol. VII.] NOVEMBER, 1860. [No. 11.**

### PERIODICAL LITERATURE.

A MAN died last year, who, in all the relations of life, possessed as faultless a character, perhaps, as any one in his day and generation. He was a gentleman—a Christian gentleman; a man of great learning, of greater piety, of the most unaffected humility, and, above all, he was a minister of the Gospel, whose wide experience and power of observation, made him acquainted with the dangers and the needs of the times, to an extent beyond that of other men. With such capabilities, and from such a stand-point, the Rev. Dr. James W. Alexander said in our hearing, but a short time before his death, in reference to "The Daily Journal Poison":

"A little mineral admixture in their daily bread, a little morbific quality in their daily milk, would be justly dreaded as tending to wear away the health; *yet the daily journal* enters your doors, distilling by little and little, false, latitudinarian, and radical opinions. No marvel if you find your old age surrounded by sons who have made shipwreck of the faith. It is impossible to watch too affectionately the literature which comes into the hands of the young. If you desire them to be guarded and manly Christians, their pabulum must be truth. It is as certain of the mind as of the body, that whatever is taken into it should tend directly to its growth and strength; all that is otherwise, is noxious. Nutrition, moreover, is a gradual process, the result of repeated acts. If, then, the mind and character are to make progress, and acquire firmness, there must be not slight and occasional, but regular and extensive study of God's revealed will. Thus, by promoting knowledge of truth, and discouraging familiarity with falsehood, we may, under

God's blessing, do much to protect ourselves against abounding infidelity."

This is a "sensation" age. The "Press" has long ago become sensational, and the Pulpit is in danger of the infection. The daily journal, the weekly newspaper, the monthly magazine, and the quarterly reviews, in their strife for compelling attention, put all inventive genius to the rack, and but too often set at defiance justice, decency, and truth. The virus has entered the ought-to-be sacred precincts of the Sunday-school, of which the *Religious Intelligencer* remarks:

"There is nothing connected with the Church, in the present day, that requires more watching than those institutions which furnish the Sunday reading of our youth. The time was when the books of these libraries were selected from the religious classics of England and America. They were the approved vehicles of religious truth. Now, however, in the desire to supply the demand of teachers and scholars for variety, a large class of works has been introduced into many schools, that, to say the least, are not calculated to lead to serious thought. Indeed, we have heard of churches where the new novels form a regular supply to the library. Although matters are not quite so bad as that in general, yet the tendency of our Sunday-school libraries is more to gratify the taste of the readers, than to strengthen or instruct their religious faculties. Unless a stop is put to this evil, it must eventually undermine the foundations of truth and righteousness in the community."

The New-York *Examiner* says of "SENSATION PREACHERS:"

"There are in all our large cities a crowd of thoughtless, sensation-loving gossips, who, with mouths agape, will run after any thing or any body that promises to gratify their depraved tastes, and as the demand for an article usually induces, before long, a supply, it is natural enough that there should be men who will desecrate the pulpit, and degrade it to the low level of a harlequin's stage.

"We do not know that there is a larger proportion of this class of mountebanks in the five hundred pulpits, more or less, of New-York and its suburbs, than among the same number of pulpits elsewhere; but we do know that there are more of them than there should be. The congregations which employ such men are partly to blame for their development."

The New-York "WORLD" declares that: "Man has been variously defined by philosophers as a cooking animal, a borrowing and lending animal, and a lying animal; differing in the opinion of the philosophers who have thus distinguished him from the rest of the animated world, chiefly in the respect of aiding the digestion of his food by means of fire, of negotiating loans, and of mendacity. The ripening civilization of these later days permits us to add variously to these definitions. With the process of the sun, and the scarcely less illuminating processes of Messrs. Hoe & Co., man has become emphatically a reading animal.

"The truth is, he reads a monstrous deal of trash and twaddle. Nine tenths of the merely literary emanations of the periodical press come appropriately under this designation. Stories of life, such as has never been lived upon our planet, delineations of manners, which are the manners neither of gods nor men, nor of the "third estate;" salient sketches, treading close upon the verge of downright immortality; fetid exhumations of the subterranean stratum of life, exposed to the light like the sores of the leper; coarseness, ribaldry, profanity, all, however, wearing the thinnest possible cloak of decency, and assuming to convey a moral inculcation while eating at the root of morality like a worm, compose an appalling proportion of the aggregate reading of the day. The denunciation is sweeping, but one less comprehensive would be inadequate.

"The French revolution and the brief but lurid reign of terror which ensued, revealed the existence in Paris of a hideous nether stratum of life of which king, courtier, priest, physician, artisan even, had never dreamed. From the Faubourg St. Antoine, its loathsome tributaries, subsidiaries, and succursals, from every reeking lane and alley of that Mecca of civilization, opulence, and profligacy, poured forth upon the Boulevards and the Elysée herds of wild-eyed men with tangled hair like the furies, unwashed, *sans culotte*, merciless, ravenous, bloody, and drunk. They swarmed about the palaces; they invaded the churches; they raved through the wards of hospitals; they leered and grimaced at the portals of nunneries. When the deluge of blood subsided, and order came again, men looked at each other and grew pale. They were walking upon a crust beneath which surged and heaved this hitherto unsuspected wave of fire.

"Society and literature, no less than geology, have their nether, intermediate, and upper strata, each in some degree unconscious of the other, as were the Parisians, of the burrowing herds of satyrs which that social upheaval unearthed. We have here, for example, a literature devoted to a strenuous advocacy of Sabbath-breaking; a literature of infidelity, a literature of crime. The thieves' lexicon is among the works with which American bibliography has recently been enriched. In addition to these, there is a huge aggregation of print which it is difficult to classify, but the effect of which is to enervate the immature mind, to inculcate false ideas of life, and to render distasteful to the young the homely and useful pursuits which alone, in most cases, lead to happiness and honor. What youth of average gifts is content to follow the plow, or wield the scythe, or swing the hammer, after the useful and humble manner of his fathers, when he is instructed by the experience of that dashing hero of the piratical tale, that the path of glory and gold is through the demesnes of adventure; that the coast of Madagascar, and the deck of a long, low, clipper-built schooner, rakish as to masts, and with unheard-of qualities as to speed, are the fields for him instead of the hill-side or the forge! Who, among the rustic maidens singing at the spinning-wheel, or among the milk-pans and other implements incident to butter, is unaffected by the perusal of the history of that rural damsel who, eloping from such common-place occupations, became a countess, and achieved lap-dogs and diamonds, and carriages and adoration! Many and many of those leprous specters who flit along the ghastly lamp-light of Broadway, bedizened, painted, and how often hungry, despairing, sick, drunk, and dying, can, if memory and mind remain, trace to the perusal of this species of literature something of the influence which directed their footsteps down the dark road, at the end of which glooms the hospital, and happily a grave without a name. The good and solvent citizen, who daily reads his mild, unexceptionable newspaper; the lettered amateur who, in the snuggest of studies, over the most fragrant of all the varieties of Souchong, beside the most cheerful of sea-coal fires, luxuriously cuts the leaves of his favorite quarterly, do not perhaps dream that the same press which conveys to them its periodical missives of instruction and recreation, conveys to multitudes that intellectual drivel which enfeebles the mind as narcotics and stimulants do the body. It is true none the less, and that portion of the press which labors in the interest of morality and religion, is recreant to its duty if it ignores it, or fails in its censure and condemnation.

"Unhappily, this vicious literature is an evil almost without remedy. Whatever the public taste demands will be supplied. Literary censorship has of course never taken root in this country, if we except its temporary grapple upon the stony soil of early puritanism, and there is, consequently, no relief except in the moral and religious sense of the community. Something may be done by the schools in fostering a taste for the better sort of light literature, something by the heads of families in rigorously excluding from the household publications bearing a shadow of taint, much by the pulpit, and much by the press. But we shall not see the end of this pernicious influence until a higher degree of education and culture takes the place of that flippant superficiality which has given us, as a people, the name of knowing less of any thing, and more of many things, than any race in the world.

"Benjamin Franklin tells us, in one of his letters, that when he was a boy, a little book fell into his hands, entitled, *Essays to do Good, by Cotton Mather.*

"It was tattered and torn, and several leaves were missing. 'But the remainder,' he says, 'gave me such a turn of thinking as to have an influence on my conduct through life; for I have always set a greater value on the character of a doer of good than any other kind of reputation; and if I have been a useful citizen, the public owes all the advantages of it to the little book.' Jeremy Bentham mentions that the current of his thoughts and studies was directed for life by a single phrase that caught his eye at the end of a pamphlet: 'The greatest good of the greatest number.' There are single sentences in the New Testament that have awakened to spiritual life hundreds of millions of dormant souls. In things of less moment, reading has wondrous power. George Law, a boy on his father's farm, met an old unknown book, which told the story of a farmer's son who went away to seek his fortune, and came home, after many years' absence, a rich man. From that moment George became uneasy, left home, lived over again the life he had read of, returned a millionaire, and paid all his father's debts. Robinson Crusoe has sent to sea more sailors than the press-gang. The story about little George Washington telling the truth about the hatchet and the plum-tree, has made many a truth-teller. We owe all the Waverley novels to Scott's early reading of the old traditions and legends; and the whole body of pastoral fiction came from Addison's sketches of Sir Robert de Coverley in the *Spectator*. But illustrations are numberless. Tremble, ye who write, and ye who publish writing. A pamphlet has precipitated a revolution. A paragraph quenches or kindles the celestial spark in a human soul—in myriads of souls."

Let parents also tremble in view of the responsibility which rests upon them, not only in preventing their children from improper reading, but also in providing them with what will attract by its beauty, instruct by its truth, and compel conviction by its point and power; or which, by its admirable simplicity, and the sweetness of its sentiments, shall mold the character for high usefulness in life, and the society of the blessed beyond the grave.

As a pioneer in this kind of reading for families, the JOURNAL OF HEALTH claims the support of the reflecting and the good. Let such deliberately read every article in any of the numbers, and then ask the following question: Is there a monthly publication in the world so free of fiction, so full of truth, so varied in its subjects, and so perfectly free from every thing calculated to offend the religious sentiment of any man who makes the Bible his rule of faith and practice, while, at the same time, it is not, technically, a religious periodical? Then comes the practical inquiry: Do I know of any safer publication, one which contains so much that is good in an equal space and at so small a cost? If not, why not order it for your child on the instant? A great name once said: "Tell me who are a man's associates, and I will tell you what kind of a man he is." Not less true is it that the character of a man is molded by his reading, and if that is truthful and pure, and of a high moral tone, there comes of it pure men and women, healthful in body, the mind cultivated, and the character spotless.

